# Not just a witness: Highlighting the utility of witness social defeat stress for the examination of neuroimmune-cardiovascular interactions across diverse populations

**DOI:** 10.1016/j.ynstr.2025.100751

**Published:** 2025-08-12

**Authors:** Margherita Barbetti, Cora E. Smiley, Monia Savi, Andrea Sgoifo, Susan K. Wood, Luca Carnevali

**Affiliations:** a Department of Chemistry, Life Sciences and Environmental Sustainability, University of Parma, Parma, Italy; b Department of Pharmacology, Physiology, and Neuroscience, University of South Carolina School of Medicine, Columbia, SC, 29209, USA; c WJB Dorn Veterans Administration Medical Center, Columbia, SC, 29209, USA; d University of South Carolina Institute for Cardiovascular Disease Research, Columbia, SC, USA

**Keywords:** Witness stress, Neuroimmune, Autonomic, Heart, Sex differences

## Abstract

Exposure to stress has widespread pathological consequences in terms of neuropsychiatric disorders and cardiovascular disease. Psychosocial stressors represent the most highly impactful and commonly experienced form of stress and, in preclinical studies, have been found to induce distinct overlapping immune and cardiovascular alterations. Historically, the social defeat model has been fundamental in providing insights into the autonomic and neuroimmune mediators of cardiovascular dysfunction in the face of social stress exposure. However, this procedure relies on aggressive, physical interaction between rodents and is limited by its almost exclusive application to young adult males. This challenges the possibility of using social defeat to generate data in rodents that can be translated into social stress-related processes in both men and women across the lifespan. More recently, a novel vicarious social defeat procedure has been developed, wherein a rodent bears witness to an aggressive social defeat encounter between two males from the safety of an adjacent compartment. This review first discusses the existing data regarding stress-induced cardiovascular alterations and the underlying autonomic and neuroimmune mediators of social defeat while critically discussing the limitations of this model. New prospects are then offered based on recent findings across a diverse population of rodent species, sexes, and ages to support the use of vicarious/witness social defeat model as an optimal strategy to investigate social stress-related autonomic, neuroimmune, and cardiovascular processes using more comprehensive and inclusive methods.

## 1 Introduction

In humans, there is accumulating epidemiological evidence regarding the link between lifetime stress exposure and increased cardiovascular disease (CVD) risk (Dar et al., 2019; Levine, 2022; Rosengren et al., 2004). As the statistics on perceived stress among the general population are staggering and continue to grow in our modern society (American Psychological Association, 2023), it is increasingly important to identify targets for treatment and prevention in both healthy individuals and patients with pre-existing CVD.

Rodent research is instrumental in improving our understanding of the direct impact of acute and chronic stressors on the integrity of the cardiovascular system and the underlying pathophysiological processes. Among the numerous stress paradigms developed for this purpose, the use of social, rather than purely physical, stressors enhance the translational value of preclinical models (Koolhaas et al., 2017). This is based on the ethological nature of such procedures, since social structure and environment can be highly significant sources of stress for many animal species, including humans and rodents, whereas laboratory stress procedures such as immobilization or electric shocks are unlikely in the evolutionary history and everyday life of these species (Sgoifo et al., 2005). Historically, the most used social stressor in rodents is the social defeat model, which is based on the classic resident-intruder paradigm (Miczek, 1979). Originally developed as a tool for preclinical aggression research in a semi-natural laboratory setting, this paradigm has been successfully adopted for the investigation of social stress-induced cardiovascular changes indicative of enhanced CVD risk predominantly in young adult male rodents. Importantly, this model has also been instrumental for providing insights into the underlying autonomic and neuroimmune mediators of stress response. However, the social defeat model does not come without limitations, such as the difficulties in discriminating between its physical and psychological aspects and in adapting it to diverse populations of rodents. To overcome some of these limitations, recent studies have established a witness/vicarious component, in which a rodent bears witness to an aggressive social defeat encounter between a male resident and a male intruder from the safety of a compartment within the resident's home cage. As such, the witness experiences the sensory and psychological aspects of an aggressive interaction between two males without risk of physical harm (Finnell et al., 2018; Iñiguez et al., 2018; Patki et al., 2014; Warren et al., 2013).

In this review, we critically discuss rodent studies of social defeat which have provided invaluable insights into the neuroimmune mechanisms underlying social stress-related cardiovascular dysfunction. Further, we offer support for the use of “witness social defeat” (WSD) as an ideal strategy for investigating the link between social stress-mediated neuroimmune signaling and related CVD risk in rodents in a comprehensive and sex-inclusive way.

## 2 The social defeat model: a history of success and missed opportunities

Social defeat is achieved in murine species by introducing the experimental animal (i.e., the intruder) to the territory (i.e., home cage) of a conspecific dominant male (i.e., the resident). The intruder animal is socially defeated through the repetition of threatening behaviors and physical attacks by the resident and displays a clear behavioral pattern of social subordination (Koolhaas et al., 2013). Following the duration of the physical encounter, a subsequent sensory component of threat is often induced by placing the aggressive resident in close proximity to the intruder, but with physical separation between them (Koolhaas et al., 2013). Inspired by the original version described by Miczek (1979), many laboratories have adopted the resident-intruder paradigm to study social stress-related physiological responses and pathological processes in rodent models. Despite the differences in experimental procedures among these studies (e.g., number of social defeat episodes, duration of physical and sensory exposure to the resident, extent of physical injury of the intruder), which have been reviewed by others (Hollis and Kabbaj, 2014), repeated social defeat (RSD) exposure consistently induces stress-related alterations in behavior, neuronal activation, endocrine signaling, and physiology (Hammels et al., 2015). For example, defeated male rodents display depressive- (e.g., anhedonia and passive coping behaviors) (Barbetti et al., 2024a) and anxiety-like behaviors (Crawford et al., 2013; Yashiro and Seki, 2017), along with neuroendocrine changes, such as elevated corticosterone levels (Carnevali et al., 2015). RSD also leads to significant brain alterations, with socially defeated male rodents exhibiting synaptic loss and structural damage in the medial prefrontal cortex (Wang et al., 2023), reduced hippocampal neurogenesis (Van Bokhoven et al., 2011), and neuroinflammatory responses (Wohleb et al., 2014). These findings are particularly relevant given the high rates of comorbidity between stress-induced cardiovascular dysfunction and neuropsychiatric disorders, which suggest overlapping underlying mechanisms capable of impacting both the brain and heart (Davidson et al., 2018; Song et al., 2019). This section will discuss the specific cardiovascular and neuroimmune changes induced by exposure to RSD as well as explore the parameters of this model that limit its application to a narrow subset of preclinical studies.

### 2.1 The cardiovascular burden of repeated social defeat stress in male rodents

The use of social defeat as a tool to study stress-related CVD risk was stimulated by the findings that a single exposure to this paradigm provokes larger cardiovascular responses (i.e., tachycardia, vagal withdrawal, and hypertension) and a greater incidence of cardiac arrhythmias compared to non-social stressors (Sgoifo et al., 1999). Further, this social stressor has been shown to precipitate markers of cardiac hypertrophy in male rodents (Costoli et al., 2004; Wideman et al., 2013). Building upon these initial findings, researchers have begun to expose male rodents to repeated episodes of social defeat stress to investigate the neurobiological and neuroimmune alterations that may lead to cardiovascular changes indicative of enhanced CVD risk.

The earliest studies on the cardiovascular effects of RSD in rodents were reviewed in 2014 (Sgoifo et al., 2014). Briefly, these studies reported that male mice exposed to daily 5-min episodes of social defeat and chronically maintained under the threat of further aggression by the continuous presence of the aggressor in an adjacent compartment for two weeks developed fibrotic tissue accumulation in the left ventricular myocardium (Costoli et al., 2004). When a similar social defeat protocol was applied to mice lacking the serotonin 1 A receptor, which has been implicated in cardiovascular stress homeostasis (Nalivaiko and Sgoifo, 2009), 27 % of the mice died from cardiac arrest (Carnevali et al., 2012). In male Sprague–Dawley rats, seven 30-min episodes of social defeat provoked a reduction in resting measures of heart rate variability (HRV), a surrogate measure of cardiac vagal modulation (Wood et al., 2012). This result was replicated in a later study in the same rat strain in which reduced HRV was associated with reduced baroreflex gain and persisted for at least six days after exposure to four consecutive days of social defeat, each consisting of three/four 10-s physical confrontations with the dominant rat (Sévoz-Couche et al., 2013). Importantly, reduced vagally-mediated HRV and baroreflex sensitivity have been shown to be predictive of life-threatening arrhythmia and death from cardiovascular causes both in humans and animal models (Billman et al., 1982; La Rovere et al., 2001; Thayer et al., 2010). Supporting these data, the assessment of the electrophysiological properties of the left ventricular myocardium of male Wild-type Groningen rats repeatedly exposed to social defeat (twelve episodes over a 25-day period) revealed the presence of (i) a decrease in the conduction velocity of the electrical wavefront, (ii) a shortening of the effective refractory period, and (iii) an increase in myocardial excitability, all of which are considered important determinants of arrhythmogenesis (Carnevali et al., 2013).

More recent studies have provided further support for the association between cardiac autonomic imbalance (i.e., low vagal modulation) and pro-arrhythmic remodeling of the rodent heart after different RSD protocols. For example, persistent autonomic alterations (i.e., lower vagally-mediated HRV) and a larger vulnerability to spontaneous ventricular arrhythmias were found in socially defeated male Sprague-Dawley rats, and particularly in a sub-population characterized by lower serum levels of brain derived neurotrophic factor (Brouillard et al., 2019, 2020). A larger vulnerability to cardiac arrhythmias after RSD (eight daily 30-min episodes) was also described in response to a pharmacological challenge with the beta-adrenergic agonist isoproterenol in male Wild-type Groningen rats (Andolina et al., 2021). In this rat strain, the same RSD protocol was also found to provoke defects in the mechanical properties of the heart both at the cellular (Barbetti et al., 2022) and organ (Andolina et al., 2021) level. Specifically, the decline in cardiomyocyte contractile performance was ascribed to signs of intracellular calcium derangement and impaired mitochondrial bioenergetic function (Barbetti et al., 2022). In another study, male Wistar rats were classified as susceptible or resilient after seven daily episodes of RSD followed by a social interaction test (Morais-Silva et al., 2019). Interestingly, susceptible rats showed depressive-like behaviors which were associated with resting tachycardia, decreased vagally-mediated HRV, and a less effective baroreflex, while resilient animals were protected from behavioral alterations and showed increased cardiac vagal modulation (i.e., higher vagally-mediated HRV) (Morais-Silva et al., 2019).

**Fig. 1:**
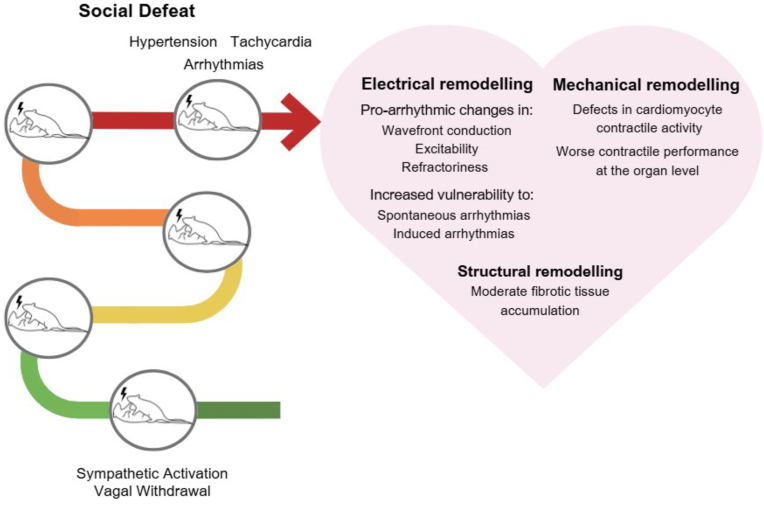
The cardiovascular burden of RSD stress exposure in young adult male rodents. Note that the number of social defeat episodes is not indicative of the severity of cardiac remodeling.

Collectively, these studies strongly indicate that different protocols of RSD stress provoke adverse remodeling of the electrical, mechanical, and structural properties of the male heart that would lead to an increased CVD risk (Fig. 1), and suggest that cardiac vagal withdrawal, indicated by reduced HRV, may represent one of the main pathophysiological mediators in vulnerable subjects.

### 2.2 Neuroimmune-cardiovascular interactions underlying the impact of RSD

Influential reviews have discussed the bidirectional relationship between the autonomic nervous system and the immune system as integral component of the CVD continuum (Carnevale, 2022; Tracey, 2002). For example, human studies suggest the presence of an inverse relationship between HRV metrics and plasma levels of inflammatory markers both in healthy subjects and patients with CVD (Haensel et al., 2008; Williams et al., 2019). Specifically, as levels of C-reactive proteins become elevated, vagal tone is reduced (Madsen et al., 2007). RSD in rodents has been established as a potent immune stimulus that is associated with increased incidence of many psychiatric and cardiovascular disorders (Dar et al., 2019; Smiley and Wood, 2022). Thus, these models have been used to shed light on the specific mechanisms of crosstalk between the autonomic and immune systems that could promote CVD under stressful conditions.

Microglia, the innate immune cells of the brain, are responsible for the neuroimmune response to RSD through the release of the proinflammatory cytokine IL-1β across many brain loci responsible for the stress response (McKim et al., 2018). One such brain region that displays increased immune signaling in response to RSD and plays a direct role in the behavioral and autonomic response to stress is the locus coeruleus (LC)-norepinephrine (NE) system (Wood et al., 2017; Wood and Valentino, 2017). Stress-induced IL-1β release in the LC has a unique ability to increase LC neuronal activity and resulting noradrenergic output (Borsody and Weiss, 2002). As the main source of central NE, the LC projects to the dorsal motor vagal nucleus (DMV) for inhibition of parasympathetic output (Ter Horst et al., 1991) and sympathetic activation (Wood and Valentino, 2017), resulting in increased heart rate and blood pressure in response to stress (Wann et al., 2010). Further, increased levels of circulating immune factors such as IL-6 and IL-1β in response to social defeat are associated with increases in LC-NE utilization and elevated atrial and brain natriuretic peptides in cardiac ventricles, proteins associated with ventricular dysfunction, and cardiac hypertrophy (Wann et al., 2010). In fact, it is well established that RSD-induced release of proinflammatory cytokines can lead to increased synthesis of brain natriuretic peptide and impairments in cardiovascular functioning (Bozkurt et al., 1998). Socially defeat males also exhibit deficits in cardiac levels of connexin-43, a gap junction protein that maintains cardiomyocyte electrical conduction and, when impaired, is associated with higher rates of ventricular arrhythmias (Foertsch et al., 2019; Pope and Wood, 2019). Importantly, these alterations in cardiac cell integrity were affected by the inflammatory cascade released in response to psychosocial stress exposure (Foertsch et al., 2019). Overall, immune activation as a result of RSD interacts with LC-NE systems as well as directly with heart tissues to alter cardiovascular functioning and leads to changes in protein expression associated with multiple facets of CVD.

### 2.3 Limitations of the social defeat model and unexplored issues

Although the stress of social defeat is mainly of a psychosocial nature, there is a potential limitation for this model due to the confounding impact of harm and injury. In a typical resident-intruder interaction, physical harm is limited (Koolhaas et al., 2013). The resident animal usually bites the back and flanks of the intruder, which are body areas with thick and tough skin (Blanchard et al., 2003). In fact, skin damage provoked by biting is typically mild and does not require any veterinary care. Moreover, the original paradigm has been refined by some investigators in different rodent species by, for example, limiting the duration and intensity of physical contact between the resident and the intruder, and extending periods of sensory exposure between them (Azzinnari et al., 2014; Kudryavtseva et al., 1991; Rygula et al., 2008). However, despite these rigorous methodological efforts, one cannot completely exclude the possibility that pain and/or inflammatory responses may be caused by this physical interaction, even when minimizing the chance of physical contact between the two confronting animals. These confounds challenge the possibility of generating information in rodents that can be translated into social stress-related processes in humans that are psychologically and emotionally rooted.

Another critical limitation of this model is the lack of ability to compare the cardiovascular effects of RSD stress between male and female rodents. For a long time, CVD has been seen as a “male” disease, due to men's higher absolute risk compared with women. However, women have recently been shown to be more vulnerable to the adverse effects of psychosocial factors on CVD, both in terms of earlier onset of the disease as well as more negative health outcomes if the disease is already present (Vaccarino and Bremner, 2017). In addition, findings of differential stress responses between male and female rodents across several dimensions highlights the importance of including both sexes when studying the effects of social stress on cardiovascular function (Dalla et al., 2005; Hodes et al., 2015; Kuske and Trainor, 2022; Sachs et al., 2014). Importantly, pregnancy alone is often considered a woman's first cardiac stress test due to the unique cardiovascular demands of pregnancy (Facca et al., 2018). As much as 20 % of women report experiencing at least three psychosocial stressors during pregnancy, with stress exposure dose-dependently corresponding to maternal morbidity (Lawrence et al., 2022). Notably, postpartum CVD affects 10–26 % of patients and includes myocardial infarction, cardiomyopathy, coronary artery disease, and hypertension (Smith et al., 2019). Yet, despite this need for research, rodent studies investigating CVD-related risk in females exposed to social stress are scarce. One possible explanation for this knowledge gap lies in the difficulty of applying the social defeat model in females, given the reluctance of male residents to attack female intruders under standard laboratory conditions. To overcome this limitation, several alternative models of social stress in female rodents have been proposed in recent years. Models of social isolation occupy a special place among these. However, while prolonged isolation has been shown to induce depressive- and anxiety-like behaviors (Brenes et al., 2020; Carnevali et al., 2020b; Liu et al., 2020; Zhao et al., 2022), its efficacy as a social stress model for studying CVD-related aspects has been questioned by studies reporting small engagement of cardiac sympathetic modulation and no significant endocrine changes in isolated animals (Benton and Brain, 1981; Carnevali et al., 2018; Smolensky et al., 2024). Additional social stressors include social crowding and social instability, the latter consisting of phases of isolation and crowding with rotation of cage mates (Haller et al., 1999; Herzog et al., 2009). However, these conditions are generally well tolerated by females (Brown, 1995). Therefore, these models might be suboptimal for studying social stress-induced cardiovascular changes in rodents of both sexes exposed to the same social stressor.

However, it must be acknowledged that in certain species or under specific conditions, social defeat can be observed in female rodents. For instance, highly territorial Syrian hamster males attack female intruders (Huhman, 2006), and female-to-female aggression has been described in highly aggressive lactating rats (Shimamoto et al., 2015; Ver Hoeve et al., 2013) and in the Peromyscus mouse species (e.g., California mice) (Steinman and Trainor, 2017; Trainor et al., 2013). More recent efforts to use the RSD model in female mice rely on the odorants and pheromones in male urine, which is applied to female intruders to increase male aggressive behavior towards them (Harris et al., 2018; van Doeselaar et al., 2021), or on manipulation of the medio-basal hypothalamus to induce female-female aggression or male-female aggression (Takahashi et al., 2017). Other strategies involve pairing ovariectomized CD-1 female mice with male partners (Kim et al., 2022), or housing intact Swiss Webster females with castrated mice (Newman et al., 2019), which can elicit aggression towards other females. Although these creative adaptations of the social defeat model have been shown to effectively establish a stress phenotype in females, these approaches involve considerable technical and logistical challenges and may compromise the ethological validity of the paradigm. Moreover, none of these studies have examined the effects of RSD on cardiovascular function in females.

Unexplored areas of social defeat research include also the investigation of the cardiovascular effects of RSD in different age groups. Indeed, several studies using social defeat in juvenile rodents have investigated the behavioral consequences in both the short- (Iñiguez et al., 2014; Mouri et al., 2018; Shimizu et al., 2020; Zhao et al., 2025) and long-term (Buwalda et al., 2013; Garcia-Carachure et al., 2023; Novick et al., 2013; Zhang et al., 2016), but none of these studies have evaluated CVD changes in this age group. In fact, social defeat studies focusing on CVD-related aspects have been conducted almost exclusively in young adult male rodents. One may, therefore, question the extent that these results may hold true for different age groups and between the sexes (Warren et al., 2020). Age plays a vital role in the deterioration of cardiovascular functionality, resulting in an increased risk of CVD in older adults (North and Sinclair, 2012). Quite surprisingly, rodent research has so far neglected the study of the age-dependent consequences of social stress on cardiovascular function, focusing only on the behavioral and neurobiological effects of social defeat in rodents of different ages. For example, some studies have compared the impact of repeated social stress exposure in adolescent rodents on adult behavioral phenotypes (e.g., Mancha-Gutiérrez et al., 2021; Mancini et al., 2023), but not on cardiovascular function. Likewise, other studies have revealed that aged mice are more vulnerable to the behavioral effects of chronic social defeat compared with younger mice (e.g., Oizumi et al., 2019), but information on cardiovascular effects is lacking.

In the next section, we propose that the WSD model may represent an ideal strategy to address the limitations and unexplored issues of social defeat stress, offering the opportunity to investigate the neurobiological mechanisms underlying stress-related CVD risk in diverse rodent populations.

## 3 Is physical interaction required? Implementation of the WSD model

The “witness social defeat” or “vicarious social defeat” model, as it has been invariably called, was originally developed in mice as a strategy to tease apart the physical and psychological components of social defeat (Warren et al., 2013). In this experimental paradigm, a rodent witnesses the physical defeat of a conspecific from the safety of an adjacent compartment within the resident's home cage (Sial et al., 2016). Typically, the witness is confined behind a clear perforated Plexiglas divider which allows visual, olfactory, and auditory perception of confrontation. Therefore, the term “witness” refers to all sensory stimuli associated with the vicarious experience of social defeat and not visual stimuli alone. Several studies have demonstrated that the repeated experience of WSD provokes the emergence of physiological (e.g., reduced body weight gain), neuroendocrine (dysregulated hypothalamic-pituitary-adrenal axis activity), and behavioral (anxiety-like and depressive-like behaviors) stress-related phenotypes in both juvenile and adult mice and rats of both sexes (reviewed in Carnevali et al., 2020a; Sial et al., 2016; Warren et al., 2020). Over the past decade, there has been a significant increase in studies investigating the consequences of WSD in diverse rodent populations. These recent investigations have further emphasized the broader applicability of this model, such as to juvenile and female rodents, contributing to its validation. Across developmental stages in both female and male rodents, WSD has been shown to induce not only anxiety-and depressive-like behaviors (e.g., reduced time spent in the open arms of the elevated plus maze test or decreased grooming in the splash test) (Kochi et al., 2017; Martínez-Caballero et al., 2024), but also disruptions of social behavior (e.g., reduced sociability) (Iñiguez et al., 2018; Yoshioka et al., 2023), increased social vigilance (Duque-Wilckens et al., 2020), as well as heightened preference for cocaine and fentanyl (Martínez-Caballero et al., 2024; Franco et al., 2022) and increased alcohol consumption (Decker Ramirez et al., 2024). These alterations resemble core symptoms of several stress-related pathologies, allowing the study of the mechanisms linking social stress exposure to pathological outcomes (Duque-Wilckens et al., 2020; Garcia-Carachure et al., 2020; Liu et al., 2024; Smiley et al., 2025).

Remarkably, these stress-related phenotypes are similar to those of conspecifics that physically experienced social defeat. For instance, both juvenile and adult rodents exposed to either RSD or WSD exhibited similar behavioral impairments, such as reduced social interaction (Garcia-Carachure et al., 2020; Kim et al., 2025), impaired contextual fear memory and extinction (Kim et al., 2025), spatial memory deficits (Kim et al., 2025; Kochi et al., 2017), as well as traditional depressive- (e.g., increased immobility in the forced swim test) and anxiety-like (e.g., reduced time in the open arms on the elevated plus maze test) behaviors (Kochi et al., 2017). Moreover, when the acute cardiovascular responses to WSD were compared, within the same cage, to the physical exposure of social defeat in male adult Sprague-Dawley rats, witnesses exhibited nearly identical pressor and tachycardic responses to that of intruders (Finnell et al., 2017). Further, neither witnesses nor intruders showed cardiovascular habituation to their respective social stress conditions upon repeated exposures (Finnell et al., 2017). Together, these studies in males suggest that WSD is a purely psychosocial stressor which removes the confounds caused by the physical interaction with the resident that occur in RSD.

### 3.1 The cardiovascular burden of repeated WSD stress

**Fig. 2:**
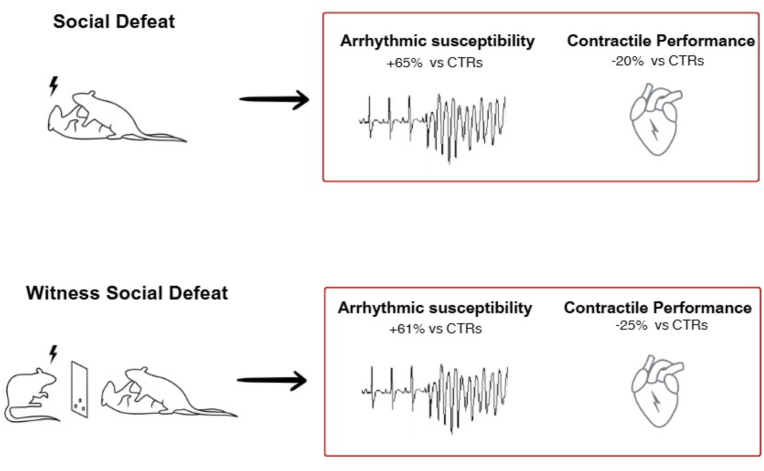
Effects of repeated episodes of social defeat versus WSD on the electrical and mechanical properties of the heart of male Wild-type Groningen rats. CTRs = controls. Percentages were based on data reported in Table 1 and refer to the arrhythmic burden and myocardial performance index, respectively.

Findings of robust stress responses during the vicarious experience of social defeat in rats have prompted the preliminary assessment of the cardiovascular burden of repeated exposures to WSD. In male Sprague-Dawley rats, witnesses exhibit significant elevations in resting systolic blood pressure without concomitant heart rate changes after five daily exposures (Finnell et al., 2017). This finding may indicate impaired baroreflex sensitivity, similarly to what was previously described in socially defeated rats (Sévoz-Couche et al., 2013). Witness male rats also showed robust increases in plasma levels of Timp-1 (tissue inhibitor of metalloproteinases-1), which in humans have been associated with indices of adverse cardiovascular remodeling and are putative indicators of increased incidence of major cardiac events (Hansson et al., 2009; Sundstrom et al., 2004). Further, in a pilot study conducted in male Wild-type Groningen rats exposed to eight episodes of WSD, we found similar consequences on the electrical and mechanical properties of the heart between witnesses and intruders. Specifically, witnesses and intruders showed a similar increased vulnerability to pharmacologically (i.e., isoproterenol) induced arrhythmias and a similar decline in the contractile performance of the myocardium compared to control rats (Fig. 2 and Table 1). These preliminary findings support the view that the cardiovascular burden of repeated WSD in male rats is comparable to that of social defeat, but without the potential confounds related to the physical interaction with the resident animal.

**Table 1:** Vulnerability to pharmacologically induced cardiac arrhythmias and hemodynamic parameters in male control, intruders, and witness rats after a RSD protocol.

	Controls (n = 7)	Intruders (n = 7)	Witnesses (n = 7)
Arrhythmic burden	54.1 ± 11.0	89.4 ± 14.9	87.3 ± 10.3
+dP/dtmax (mmHg/s)	7731 ± 87	6830 ± 168	6111 ± 129
-dP/dtmax (mmHg/s)	−6566 ± 202	−6197 ± 212	−5302 ± 109
Isovolumic contraction time (s)	0.0180 ± 0.0003	0.0210 ± 0.0006	0.0220 ± 0.0004
Myocardial performance index	0.46 ± 0.01	0.55 ± 0.02	0.57 ± 0.03

*Notes.* Data are reported as mean ± SEM. Data of control and intruder rats refer to those published in our previous study (Andolina et al., 2021). Data of witness rats were obtained from a pilot study conducted in our lab in similar experimental conditions. All rats belonged to the wild-type Groningen strain and were age-matched. Vulnerability to pharmacologically (i.e., isoproterenol) induced cardiac arrhythmias and hemodynamic parameters were evaluated 24 h and 72 h after a RSD protocol, respectively. Detailed experimental procedures and analyses are reported in the original study (Andolina et al., 2021). The peak rate of rise (+dP/dtmax) and decline (-dP/dtmax) of left ventricular pressure, and the isovolumic contraction time are indexes of myocardial efficiency. The myocardial performance index reflects global myocardial function and possesses prognostic value in several pathologic conditions, with higher values indicating worse performance (Salemi et al., 2004). Please note that these data are simply descriptive, and no statistical comparisons of the three groups are reported (statistical comparisons of intruders versus controls are reported in the original paper, (Andolina et al., 2021).

**Fig. 3:**
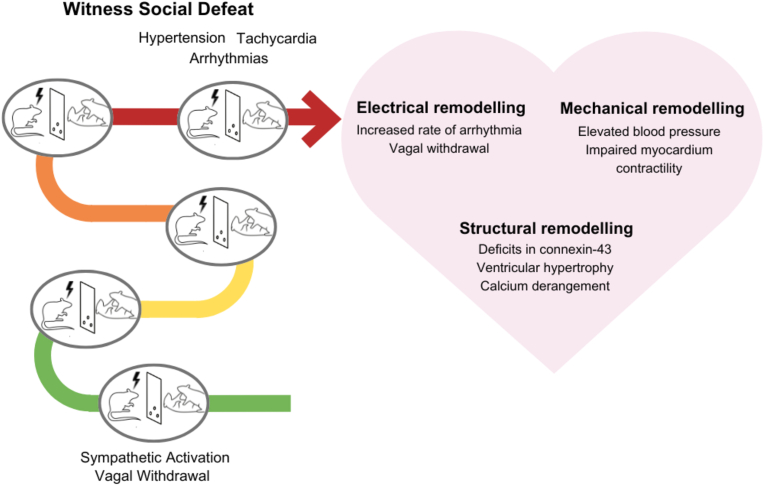
Cardiovascular impact of acute and repeated WSD in adult female rodents.

Importantly, witnessing the social defeat bout of a male conspecific elicited remarkable pressor and tachycardic responses also in female Sprague-Dawley rats (Finnell et al., 2018; Pate et al., 2023). Specifically, with acute (one day) and repeated (five days) WSD, adult females exhibit higher mean arterial pressure, heart rate, ventricular weight, and an increased number of arrhythmias, particularly premature ventricular contractions (PVCs). Repeated WSD in females also affects the sympathovagal balance, with vagal withdrawal observed in these animals, indicative of a greater sympathetic response and reduced capacity of the parasympathetic system to cope with stress (Finnell et al., 2018). Additionally, this impairment in vagal tone observed in female witnesses may underlie the increased incidence of PVCs, since normal vagal function provides a protective effect against tachyarrhythmias. Of note, these cardiovascular responses in female witnesses were found to be independent of the specific estrous cycle stage yet dependent on the presence of ovarian hormones, as removal of the ovaries prevented these alterations (Finnell et al., 2018). Further, a study in the female Wild-type Groningen rat strain revealed that nine daily episodes of WSD provoked the emergence of behavioral and neuroendocrine stress-related phenotypes, which were associated with an impairment of the contractile properties of isolated cardiomyocytes and prolonged intracellular Ca^2+^ derangement in adult females (Barbetti et al., 2023). These contractile defects were ascribed to potential alterations in the conformational equilibrium of sarcoplasmic reticulum Ca^2+−^ATPase/phospholamban complex induced by repeated witness stress (Barbetti et al., 2023). Essentially, the machinery involved in cardiac relaxation exhibits impairments following repeated WSD in females, which may underlie the alterations in cardiovascular functioning observed across the literature (Barbetti et al., 2023; Finnell et al., 2018). Overall, exposure to WSD evokes a distinct array of cardiovascular alterations in adult female rodents (Fig. 3), suggesting that the use of WSD could also inform our understanding of sex differences in the cardiovascular consequences of social stress exposure. In this regard, a recent study investigated the impact of WSD on cardiovascular function in age-matched adult wild-type Groningen rats of both sexes, which vicariously experienced the social defeat bout between two males for nine consecutive days (Barbetti et al., 2025). After repeated WS exposure, signs of cardiac electrical and mechanical impairments were found in both sexes, but to a greater extent in males compared to age-matched females. Importantly, the greater electromechanical vulnerability of the male heart was associated with specific molecular pathways involving epigenetic (miRNA)-mediated control of SIRT-1, a protein which plays a crucial role in cardiovascular physiology (Barbetti et al., 2025). These findings support the use of WSD as an optimal method of investigating preclinical stress-induced cardiovascular dysfunction in a sex-inclusive way.

### 3.2 WSD-related neuroimmune mechanisms of cardiovascular dysfunction

While exposure to stress has long been associated with the development of cardiovascular dysfunction, the neuroimmune mechanisms that may influence this disease state have only recently become a focus of study (Black and Garbutt, 2002). Recent experiments have determined that WSD is capable of affecting both central and peripheral immune activity across multiple species, sex, and age groups (Decker Ramirez et al., 2024; González-Portilla et al., 2025; Hodes et al., 2014; Martínez-Caballero et al., 2024; Nakatake et al., 2020; Smiley et al., 2025). Further, individual differences in the immune response to WSD are associated with differences in the behavioral and cardiovascular susceptibility to this social stressor (Finnell et al., 2018; Finnell and Wood, 2018). Interestingly, transplanting the immune system of a mouse exposed to WSD into a non-stressed animal rendered the latter more susceptible to the impact of stress (Hodes et al., 2014). Additional associative studies have established that WSD induces a similar cardiovascular response to RSD and initiates a similar inflammatory response (Finnell et al., 2017). The functional connection between stress-induced inflammatory signaling and cardiovascular impairment may, in part, be due to the impact of immune signaling on the major stress hormone corticotropin releasing factor (CRF) (Black and Garbutt, 2002). Stress-induced cytokine activation stimulates the release of CRF which, in acute and chronic conditions, serves to increase blood pressure and heart rate while reducing baroreflex sensitivity, a phenotype consistent with multiple types of CVD (Chesterton et al., 2005). CRF is also capable of directly altering the activity of autonomic brain regions to impact cardiovascular function (Yamaguchi and Okada, 2009). Further, autonomic nerves have been observed to have cytokine receptors which may directly allow inflammatory signaling resulting from stress to impact cardiovascular function (Takefuji and Murohara, 2019). Overall, direct, through activation of autonomic regions, and indirect, through augmented CRF release, actions of stress-induced neuroimmune signaling are highly associated with cardiovascular dysfunction.

Recent mechanistic studies have reinforced the role of WSD-induced neuroimmune signaling in the cardiovascular impact of stress (Finnell et al., 2018; Smiley et al., 2023). Exposure to repeated WSD in female rats leads to vagal/parasympathetic withdrawal, where the high frequency component of HRV is blunted during acute and repeated WSD exposure (Pate et al., 2023). Importantly, this study identified that intra-LC infusion of clodronate, a compound used to selectively ablate microglia, inhibited WSD-evoked vagal withdrawal, and points to a neuroimmune-mediated mechanism of WSD-induced cardiovascular impairment. While the inflammatory mechanisms of WSD have been recently established, investigation into the role of neuroimmune signaling in the cardiovascular consequences of WSD is a promising new realm of study.

## 4 Conclusion and perspectives

The social defeat model has been and continues to be an invaluable tool for investigation into the neurobiological mechanisms of social stress exposure and CVD-related risk in rodent models. By applying different variants of RSD, several studies have provided important insights into the cardiovascular burden of social stress exposure and the potential neuroimmune and autonomic mediators. However, these studies were limited by the potential confounds related to the physical interaction and were mainly applied to rodent populations that were age- (young adult) and sex- (male) restricted. Therefore, many questions remain unanswered such as the age-dependent and sex-specific consequences of social defeat stress on cardiovascular function.

The emerging studies on WSD reviewed herein demonstrate that the vicarious experience of social defeat induces potent cardiovascular responses and alterations in the electrical and mechanical properties of the male heart which are similar to those of socially defeated animals, but without the potential confounds related to the physical interaction with the resident animal (e.g., pain, inflammatory responses). Further, recent evidence indicates that the adverse consequences of repeated exposures to WSD extend to the female rodent heart and are dependent on the neuroimmune impact of social stress. These findings emphasize our support for this model to be utilized for studying sex-differences in cardiovascular outcomes and underlying neurobiological and pathological processes of social stress in rodents. Indeed, studies using WSD to compare social stress consequences in rodents of both sexes have revealed sex-specific behavioral responses, including more pronounced vigilant behavior in females compared to males (Barbetti et al., 2024b; Duque-Wilckens et al., 2020). Additional sex-specific behavioral and cardiovascular alterations have been observed, which appear to be related to signaling pathways associated with mood-related disorders (Morais-Silva et al., 2023; Pagliusi et al., 2022) and with sex-specific neuronal activation (Canto-de-Souza et al., 2025) and cardiac epigenetic changes (Barbetti et al., 2025), which may contribute to sex-specific vulnerabilities to the long-term consequences of chronic social stress. With this paper, we suggest that expanding the social defeat model by adding a witness component represents a useful strategy for addressing the issue of social stress-related CVD risk in different age-groups to study stress in adolescence through aged adults, and in both sexes, including the highly sensitive peripartum period in females.

Importantly, therapeutic strategies (e.g., prolonged exposure therapy) commonly used in stress-related psychiatric disorders, such as post-traumatic stress disorder, have been shown to reverse behavioral and neurophysiological impairments in mice exposed to WSD (Kim et al., 2025), suggesting the translational relevance of this paradigm, not only as a model of social stress, but also as a platform for preclinical treatment development. Critically, important questions arise from the use of the WSD model that are widely relevant to many social stress-related fields. What contextual factors are threatening enough to invoke stress responses in witnesses? What stress appraisal and coping processes are involved? Does the degree of relatedness between the witness and intruder play a role in the magnitude of these responses? Does the outcome response of the intruder impact the witness? It must be also acknowledged from an ethical perspective that more animals are needed in a WSD protocol compared to the traditional social defeat.

In conclusion, we propose that rodent research on the neurobiological mechanism underlying social stress-related CVD risk should embrace the WSD model to inform the identification of targets for treatment and prevention in a more comprehensive and sex-inclusive way.

## CRediT authorship contribution statement

**Margherita Barbetti:** Writing – review & editing, Visualization, Conceptualization. **Cora E. Smiley:** Writing – review & editing, Visualization, Conceptualization. **Monia Savi:** Writing – review & editing. **Andrea Sgoifo:** Writing – review & editing. **Susan K. Wood:** Writing – review & editing, Funding acquisition, Conceptualization. **Luca Carnevali:** Writing – original draft, Funding acquisition, Conceptualization.

## Funding

This work was supported by #NEXTGENERATIONEU (NGEU) and funded by the Ministry of University and Research (MUR), National Recovery and Resilience Plan (NRRP), project MNESYS (PE0000006) – A Multiscale integrated approach to the study of the nervous system in health and disease (DN. 1553 October 11, 2022). Dr. Smiley was supported by the National Institute on Drug Abuse (F32 DA058380), Dr. Wood was supported by the Veteran's Health Administration (BX005661, BX002664, and BX006218), and the National Heart Lung and Blood Institute of NIH (R01HL179186).

## Declaration of competing Interest

The authors declare that they have no known competing financial interests or personal relationships that could have appeared to influence the work reported in this paper.

## Data Availability

Data will be made available on request.
